# The Natural Antioxidants, Pomegranate Extract and Soy Isoflavones, Favourably Modulate Canine Endothelial Cell Function

**DOI:** 10.5402/2012/590328

**Published:** 2012-11-26

**Authors:** Sabina M. Baumgartner-Parzer, Ferdinand Rudolf Waldenberger, Angelika Freudenthaler, Amandine Ginouvès-Guerdoux, David McGahie, Hugues Gatto

**Affiliations:** ^1^Clinical Division of Endocrinology and Metabolism, Department of Internal Medicine III, Medical University of Vienna, 1090 Vienna, Austria; ^2^Heart Center Hietzing, Wolkersbergstra**β**e 1, 1130 Vienna, Austria; ^3^Unlicensed Product Development Unit, Virbac, 13ème rue, 06511 Carros Cedex, France; ^4^Medical Department, Virbac, 13ème rue, 06511 Carros Cedex, France

## Abstract

Cardiovascular disease, preceded by vascular endothelial dysfunction, is a prominent cause of death in dogs. L-carnitine and taurine, well known for their antioxidative capacity, beneficially affect cardiovascular disease as well as certain dog cardiomyopathies. It is well established that vascular endothelial dysfunction precedes cardiovascular disease and that “vasoprotective factors” (NO and antioxidants) prevent apoptosis, whereas “risk factors” such as oxidized LDL, hyperglycemia, and free fatty acids trigger it in cultured human vascular endothelial cells. Whereas human vascular cell *in vitro* models are widely established and used for the characterisation of potential vasoprotective substances, such models are not available for canine endothelial cells. In the present study we therefore developed an *in vitro* model, which allows the testing of the effects of different substances on proliferation and apoptosis in canine aortic endothelial cells. This model was used to test L-carnitine, taurine, pomegranate extract, and Soy Isoflavones in comparison to reference substances (glutathione and pioglitazone) previously shown to modulate human endothelial cell function. L-carnitine and taurine neither exhibited antiproliferative nor antiapoptotic activities in the context of this study. However extracts from pomegranate and soy isoflavones dramatically reduced proliferation and apoptosis in a dose dependent fashion, being in line with a vasoprotective activity in dogs.

## 1. Introduction

The increased life expectancy of dogs over recent years, probably due mainly to advances in canine nutrition and health care, has also been associated with an increased prevalence of cardiovascular disease [1]. The vascular endothelium represents a widespread and interactive organ with various biological actions including barrier function, secretion of anti/prothrombotic factors, leukocyte and platelet adhesion and, very importantly, regulation of vascular tone [2, 3]. Macrovascular disease and heart failure are preceded and predicted by increased apoptosis of endothelial cells and dysfunction of the vascular endothelium including increased endothelial cell turnover to maintain an intact endothelial lining, increased smooth muscle cell migration due to an impaired endothelial barrier function, and a loss of vascular elasticity leading to an increased afterload [2, 4–6]. Moreover, it is well established that heart failure, experimentally induced in dogs by rapid pacing [7], as well as vascular endothelial dysfunction relate to reduced generation and/or exaggerated degradation of nitric oxide (NO). Reduced bioavailability of NO is associated with exaggerated superoxide anion production, increased oxidative stress, and sustained vasoconstriction [2, 8]. Such effects are believed to trigger vascular endothelial cell apoptosis and, in the long run, result in progression of human and canine valvular disease and heart failure [9–12]. Therefore, identification and characterization of vasoprotective agents and of their effects on endothelial cell function are of major importance in order to prevent or ameliorate the sequelae of endothelial dysfunction and vascular disease.

So far L-carnitine and taurine, both known for their antioxidative capacity [13, 14], have been shown to be associated with beneficial effects in human endothelial cells as well in certain dog cardiomyopathies [15–22]. Similarly, polyphenolic compounds such as isoflavones found in soybeans and tannins present in pomegranate extracts exhibit antioxidant and cyto- and cardioprotective activities [23–30]. Concerning the human vascular endothelium, it has been proven by a considerable number of studies that factors often termed “vasoprotective factors” (NO, shear stress, antioxidative agents) prevent, whereas “atherosclerotic risk factors” (oxidized/glycated LDL, hyperglycemia, proinflammatory cytokines, elevated free fatty acids) trigger apoptosis in cultured human vascular endothelial cells [31–39].

Due to a lack of such models for the canine vascular endothelium, it was largely unknown to which extent the beneficial effects of L-carnitine, taurine, soy isoflavones, and pomegranate extract for the cardiovascular system relate to direct effects of those antioxidants on canine endothelial cell health and function. A previous recent study demonstrated that they could decrease the loss of viability of canine aortic endothelial cells (CnAoEC) under conditions of oxidative stress [40]. However the viability assay used in that study relied on total metabolic activity, and therefore it could be theorised that there is a risk of artificially good results if the tested substances concurrently promote significant excessive proliferation in surviving cells. Information is therefore still lacking regarding the impact of these substances on key indicators of functional health in canine endothelial cells such as apoptosis and proliferation. Moreover, it remains to be elucidated whether the observed effects of substances known to modulate these parameters are species dependent or can be extrapolated from species to species [41]. 

Therefore, the present study aimed at development of an *in vitro* model on the basis of CnAoEC, in order to characterize the potential beneficial or detrimental effects of different test agents with respect to canine endothelial cell proliferation and apoptosis. Different substances (GSH, NAc, insulin sensitizers, etc.), previously well characterized in human *in vitro* models for endothelial dysfunction [34–38], were used as putative references for the development of our canine model and as internal controls for subsequent assays performed with test agents. We hypothesised that the reference substances would produce similar effects in CnAoEC to those seen with human endothelial cells. We further hypothesised that the previously noted beneficial effect of the four test substances [40] would be associated with stable or decreased apoptosis and proliferation, thus confirming the interest of these substances in developing a multidimensional dietary strategy to reduce the onset and progression of the canine endothelial degeneration involved in progressive valvular diseases.

## 2. Research Design and Methods

### 2.1. Reagent Sources

CnAoECs and the respective media (CECBM, HBSS) and growth supplements were purchased from Cell Applications, Inc. (San Diego, USA). Linoleic acid (LoIS), *γ*-linolenic acid (ALens), fibronectin, bovine Serum albumin (BSA), dimethyl sulfoxide (DMSO), glutathione (GSH), and N-acetylcysteine (NAc) were purchased from Sigma Chemical Co. Phosphate buffered saline (PBS) and trypsin ethylenediaminetetraacetic acid (trypsin EDTA) were from BioWhittaker/Lonza (Belgium), human vascular endothelial growth factor (VEGF) and basic fibroblast growth factor (bFGF) were purchased from BioVision, and [Methyl-^3^H] thymidine from Amersham Pharmacia. Taurine and L-carnitine L-tartrate were from Azelis Pharma (Paris, France), pomegranate extract (40% punicosides) from Polinat (Las Palmas, Spain), and soy extract (standardized at 40% soy isoflavones) from ADM (Decatur, IL, USA).

### 2.2. Test and Reference Substances 

#### 2.2.1. Reference Substances

GSH (10 mM , equivalent to 3.1 mg /mL), NAc (5 mM , equivalent to 816 *μ*g/mL), VEGF (25 ng /mL), bFGF (10 ng /mL), LoIS (50 *μ*M, equivalent to 14 *μ*g/mL), ALenS (50 *μ*M, equivalent to 13.9 *μ*g/mL), and the insulin sensitizers Pioglitazone (Pio) (50 *μ*M, equivalent to 17.8 *μ*g/mL) and Rosiglitazone (Rosi) (50 *μ*M, equivalent to 17.9 *μ*g/mL) were prepared and used as previously described [34–38]. In brief, free fatty acids (LoIS and ALenS) were dissolved in ethanol, the insulin sensitizers (Pio and Rosi) were dissolved in DMSO, all other reference substances were soluble in water. Where DMSO or ethanol were required for the test or reference substances, the respective control cultures had the equivalent concentrations of DMSO (1%) or ethanol (1.5%), respectively. 

#### 2.2.2. Test Substances

 L-carnitine, taurine, pomegranate extract, and soy extract were kept dry and protected from light (in brown flasks, covered with parafilm (bottle tops) and with aluminium foil). Before each experiment, fresh stock solutions were prepared for each substance: soy isoflavones were dissolved in DMSO (250 mg/mL), all other test substances were soluble in water (50 mg/mL). Where DMSO was required for the test substances, the respective control cultures had the equivalent concentrations of 1% DMSO. The sample concentrations to be tested in the different assays, that is, 1, 50, and 250 *μ*g/mL, were estimated by reference to existing published studies using human endothelial cells or data reporting plasma concentrations in dogs or humans following oral supplementation [15, 19, 42–44], and the absence of cytotoxicity of the proposed levels of these substances on CnAoEC (data not shown).

Experimental wells (with test or reference substances) were related to the respective control wells (without test or reference substances), set to 100%.

### 2.3. Canine Aortic Endothelial Cell Culture

CnAoECs received as cryopreserved vial containing 500 000 cells were immediately frozen in liquid nitrogen and kept there until use. For further use, cells were thawed at 37°C, resuspended in CECGM (according to the manufacturer's instructions), and cultured on Fibronectin (0.0025% in PBS) coated (2 h/37°C) cell culture dishes at 37°C/5% CO_2_. 

For subcultures, confluent cells were rinsed with HBSS and treated with trypsin/EDTA (6 mL/75 cm^2^ ), followed by addition of 8 mL trypsin neutralisation solution (5% BSA in PBS). After centrifugation (5 min/220 g) the collected cells were replated in CECGM with a density of 5 000 to 10 000 cells/cm^2^.

The day after seeding and every other day, the medium was changed and confluency of the cells monitored by phase contrast microscopy. 

For proliferation and apoptosis assays, cells were used between passages 5 and 7.

### 2.4. Proliferation Assays

**Figure 1:**
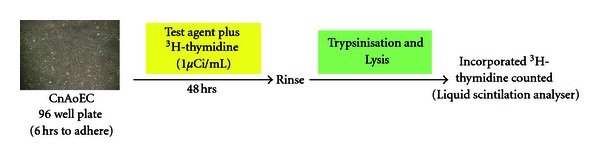
Proliferation Assay.

Proliferation assays (see Figure 1) were carried out as described previously [34, 35, 37]. In brief, confluent CnAoECs cultures were replated in flat bottomed 96-well tissue culture plates (10,000 cells per well) and allowed to adhere for 6 h . Subsequently, cells were exposed to ^3^H-thymidine (final concentration: 1 *μ*Ci/mL = 37 kBq/mL) and the respective test agents for 48 h . After two washing steps with PBS, cells were trypsinized, lysed by a freeze thaw cycle, harvested, and incorporated. ^3^H-thymidine was counted in a Tri-Carb Liquid Scintillation Analyser (Canberra Packard, Meriden, USA). Samples were tested in quadruplicates. Results of experimental cultures exposed to the test substances are presented in relation to intraindividual control cultures (without test substances). Control cultures were set to 100%. 

### 2.5. Apoptosis Assays

**Figure 2:**
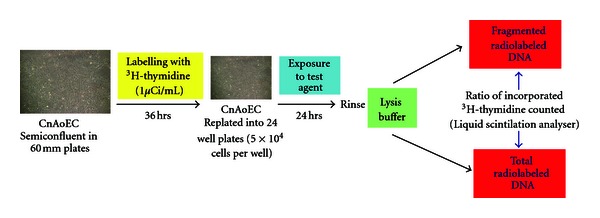
Apoptosis Assay.

Apoptosis assays (see Figure 2) were performed as previously described [34, 35, 39]. In brief, semiconfluent plates (60 mm) of CnAoECs were labelled with ^3^H-thymidine (37 kBq/mL, 36 h) and were subsequently replated into 24-well culture plates (5 × 104 cells/well). After their exposure to the test substances (24 hours) cells were treated with lysis buffer (20 mmol/l Tris.Cl, pH 7.5, and 0.4% Triton X-100 in PBS). Fragmented (apoptotic) radiolabeled DNA in the supernatant was counted in a Liquid Scintillation Analyser (Canberra Packard, Meriden, CT) and was then related to total incorporated radioactivity of cells (quantified after digestion of the remaining suspension with 180 *μ*g/mL DNase, Boehringer Mannheim, Germany). Experiments were performed in triplicates or duplicates. Results of experimental cultures exposed to test substances are presented in relation to intraindividual control cultures (without test substances). Control cultures were set to 100%. 

### 2.6. Statistics

Data are expressed as means ± SD. Statistical analysis was performed using Student's paired *t*-test.

## 3. Results

### 3.1. Proliferation

#### 3.1.1. Reference Substances

**Figure 3:**
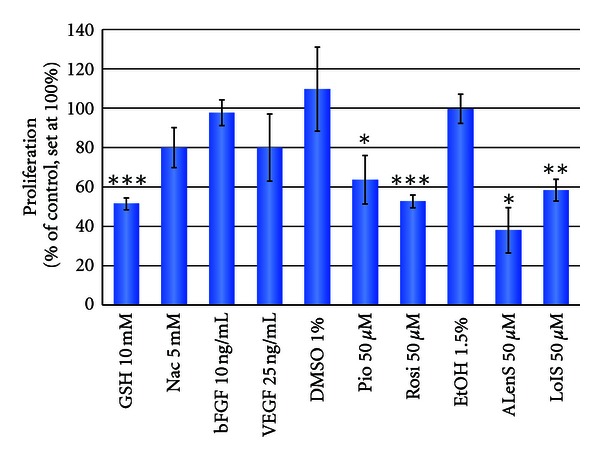
Impact of selected reference substances on the proliferation of CnAoECs (mean of 3 independent experiments each performed in 4 wells) *P < 0.05; **P < 0.01; ***P < 0.005.

As depicted in Figure 3, the antioxidant GSH, the insulin sensitizers Pio and Rosi, and the free fatty acids LoIS and ALenS significantly reduced proliferation in CnAoECs, such results being in line with previous work in different types of human vascular endothelial cells [35, 37]. Of note, however, VEGF and bFGF, known to increase proliferation in human endothelial cells [34], did not provoke such proproliferative response in canine cells.

Ethanol and DMSO, used as solvents for FFAs and insulin sensitizers, respectively, did not significantly affect CnAoECs proliferation.

#### 3.1.2. Test Substances

**Figure 4:**
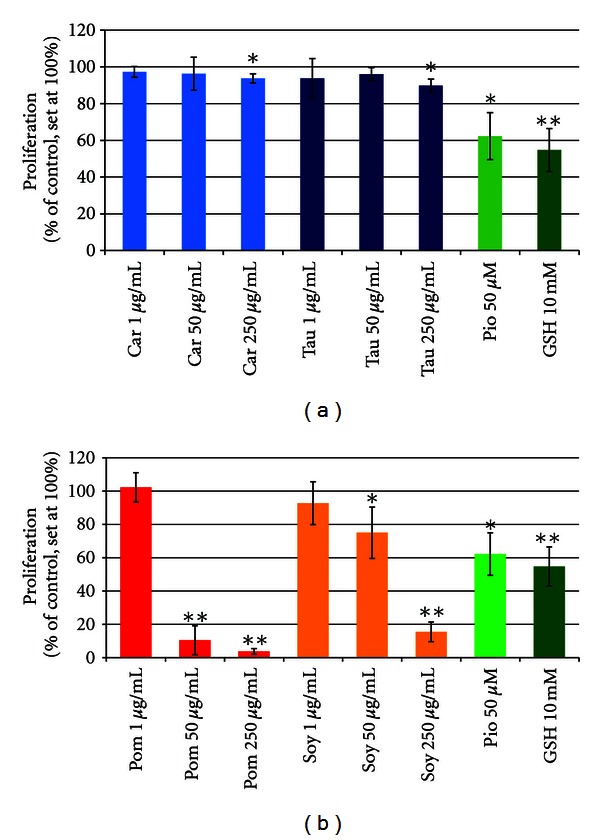
(a) Modulation of proliferation of CnAoECs by carnitine and taurine in comparison to the reference substances Pio and GSH (mean of 4 experiments, each in 4 wells) *P < 0.05; **P < 0.01. (b) Modulation of proliferation of CnAoECs by pomegranate extract and soy isoflavone extract in comparison to the reference substances Pio and GSH (mean of 4 experiments, each in 4 wells) *P < 0.05; **P < 0.01.

In CnAoECs, L-carnitine and taurine induced a slight but significant reduction of proliferation by 6.25% and 10.25%, respectively, at the highest concentration only (250 *μ*g/mL, see Figure 4(a)). However, pomegranate-extract and soy isoflavones markedly reduced CnAoECs proliferation, even at a concentration of 50 *μ*g/mL, by 90% and 25%, respectively, and in a dose-dependent manner (Figure 4(b)). Exposure of CnAoECs to 250 *μ*g/mL of these two substances led to an impressive and highly significant inhibition of the cells' proliferation to 3.75% and 15.5% of control (set to 100%), whereas Pio and GSH reduced proliferation only to 62.25% and 54.75% of control, respectively (Figure 4(b)).

### 3.2. Apoptosis

#### 3.2.1. Reference Substances

**Figure 5:**
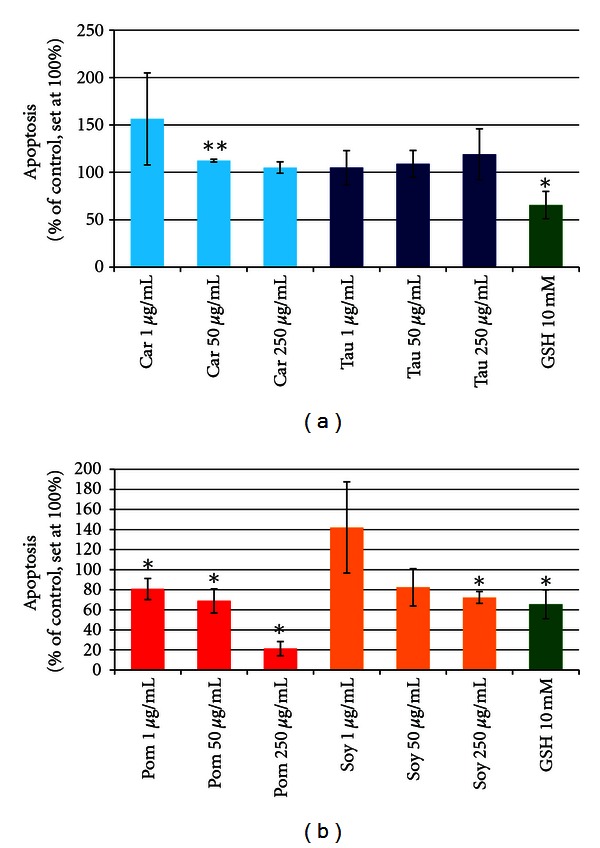
(a) Modulation of apoptosis in CnAoECs by carnitine and taurine in comparison to the reference substance GSH (means of 3 independent experiments, each in 3 wells) *P < 0.05; **P < 0.01.  (b) Modulation of apoptosis in CnAoECs by pomegranate extract and soy isoflavone extract in comparison to the reference substance GSH (means of 3 independent experiments, each in 3 wells) *P < 0.05.

As shown in Figure 5(a), the antioxidant GSH at 10 mM  markedly reduced apoptosis (by 35%) in CnAoECs. In contrast, in CnAoECs exposed to 300 *μ*mol/L LoIS and ALenS, death rates dramatically increased, more than 55% of cells having undergone apoptosis after 48 h of incubation (data not shown). Such antiapoptotic effects of antioxidants as well as the proapoptotic response exerted by free fatty acids have previously been observed in human vascular endothelial cells [34, 35].

#### 3.2.2. Test Substances

L-carnitine and taurine did not markedly affect apoptosis in CnAoECs, the statistical significance observed for 50 *μ*g/mL Carnitine being probably related to the very low standard deviation observed for that particular concentration (Figure 5(a)).

The antiapoptotic effect of soy extract was significant only for the highest concentration tested (250 *μ*g/mL). In contrast, pomegranate extracts significantly reduced apoptosis at all concentrations tested and in a dose dependent fashion, showing the most striking effect at 250 *μ*g/mL by a reduction of 73% compared to control cells (Figure 5(b)).

## 4. Discussion

Due to the increasing amount of evidence linking endothelial dysfunction to the onset and progression of heart failure in dogs [11, 12], it is important to have a method of screening and/or testing substances that may have potential to protect endothelial function *in vitro*. This is already widely done with human endothelial cells, and the markers of excessive proliferation and apoptosis are crucial indicators of dysfunction. However, until now, no such model existed using canine endothelial cells. This is why we began the study with the establishment of an *in vitro* cell culture model which allowed the reproducible analysis of these markers in response to different stimuli. This then enabled, as a second step, the identification of pro- and antiproliferative as well as of pro- and antiapoptotic agents. 

The reference substances were chosen on the basis of their abilities to exert anti- as well as proproliferative and anti- and proapoptotic responses in human endothelial cell culture models. Of note, the observed antiapoptotic and antiproliferative effects were similar in human endothelial cells and the tested canine endothelial cells for the antioxidants GSH and NAc as well as for the insulin sensitizers Pio and Rosi [34, 35, 37]. The free fatty acids exhibit marked proapoptotic action in canine endothelial cells, which clearly exceeds the effects exerted in human vascular endothelial cells [35]. Of course it must be noted that this relates to use of these particular free fatty acids in isolation, as opposed to the more normal dietary situation where a balance of Omega-3 and Omega-6 oils will be present, and where the ratio of these oils has already been shown to be important [45]. In contrast to human umbilical vein endothelial cells [34], VEGF and bFGF, were not able to provoke a proproliferative response in CnAoECs. In that context it is of note that it has previously been speculated that the effects of natural antioxidant molecules in endothelial cells can be species dependent [41]. As could be observed for the reference substances GSH and PIO, which were used as internal controls in each assay performed for the test substances, the model exhibited considerable reproducibility throughout the whole study, even if different settings (coating, growth factor supplement, etc.) were employed (data not shown). This therefore partially confirms our initial hypothesis that substances shown to be beneficial for human endothelial cells will also produce beneficial results in CnAoECs, but intriguingly suggests that it is not possible to extrapolate these results directly across species due to occasional differences in the intensity of the effects. This confirms the need to test substances in cells derived from the target species.

For the first time this study shows that extracts of pomegranate and soy isoflavones are able to exhibit dramatic antiapoptotic activities in CnAOECs. Pomegranate extract's antiapoptotic effect was already detectable at the lowest concentration tested (1 *μ*g/mL) and was dose-dependently sustained until the highest concentration applied (250 *μ*g/mL). Accelerated apoptosis of vascular endothelial cells is relevant in the development and progression of cardiovascular disease [4–6]. We have previously shown that vasoprotective agents such as antioxidants (including lipoic acid and GSH) or insulin sensitizers exhibit both antiapoptotic and antiproliferative effects in human vascular endothelial cells. Those effects could prevent loss of the endothelial barrier function (due to accelerated endothelial cell apoptosis) and exhaustion of the endothelial cells' proliferative capacity (due to accelerated proliferation). Both the antiapoptotic and antiproliferative activity of pomegranate and isoflavones in CnAoECs therefore suggest a vasoprotective action of those polyphenolic compounds in dogs, which could beneficially affect chronic mitral valvular insufficiency (CMVI), the major cause of heart failure in this species [1]. Since progression of CMVI is assumed to be triggered by endothelial dysfunction and the latter vice versa is potentiated by CMVI-associated increased cellular oxygen demand [4, 11, 12, 17, 46–48] the cytoprotective antiapoptotic as well as antioxidative effects of pomegranate [49] and soy isoflavone extracts could beneficially affect the progression of CMVI to heart failure in dogs.

Both of these agents are complex biological substances containing multiple potentially active polyphenols or isoflavones. It was not possible or appropriate to attempt to identify the balance of the effects of individual components of complex natural substances such as these, but indeed this kind of complexity using agents with a multi-faceted action has been proposed as a necessary and important part of rational use of antioxidants to prevent cardiovascular disease [50].

L-carnitine and taurine were previously shown to exert protective effects against oxidative stress in human endothelial cells [15, 22]. In a recent study we have shown that pomegranate extract, alone and in combination with soy isoflavones, taurine, and L-carnitine, also shows strong protective effects against oxidative stress in CnAoECs [40]. In that context it is of note that the survival signaling pathways of the Bcl-2 family, PI3Kinase, and estrogen receptor are assumed to mediate the protective antioxidative effects of the soy-derived isoflavones genistein and daidzein [30]. Such pathways have previously been shown to mediate antiapoptotic effects described for lipoic acid [34] in human vascular endothelial cells. Since taurine and L-carnitine, in contrast to extracts of pomegranate and soy isoflavones, did not affect apoptosis in canine aortic endothelial cells, it is tempting to speculate that different mechanisms are involved in those substances' antioxidative and beneficial vascular effects in dogs. It is also worth noting that any results obtained for extracts of pomegranate are probably specific to each type of extract. It has been shown that extracts obtained from different parts of the plant have widely varying compositions of active components [49, 51].

 Concerning the concentrations applied, there is a wide range of concentrations described in the literature for antioxidants, depending on species and compound. To cover such variety the test substances were used in a broad range from 1 to 250 *μ*g/mL. The lack of any cytotoxic effects at the highest concentrations we used also provides some reassurance regarding the potential use of these compounds.

One limitation of a study such as this is related to the use of an *in vitro* cell-based model. Indeed it is possible that there may be differences in the responses of cells in the *in vivo* situation compared to the *in vitro* environment, and it is also not possible to assess completely the impact of the diseased state on the function of the endothelial cells. One possible solution could be to use isolated segments of blood vessel to assess vasorelaxation responses. However this equally would have significant limitations and would preclude the direct assessment of key markers such as apoptosis. On balance, as substances having beneficial effects on proliferation and apoptosis of human endothelial cells in the *in vitro* situation have been shown to also provide *in vivo* benefits, this type of study is a rational step providing key information regarding the potential benefits of these substances in the canine species.

In conclusion, this is the first study showing that pomegranate extract and soy isoflavones inhibit apoptosis and proliferation in canine aortic endothelial cells. This supports our hypothesis that these agents could have a role in prevention or amelioration of clinical endothelial dysfunction and therefore progression of cardiovascular disease in dogs [47, 48]. Further studies will, however, be necessary to evaluate the underlying mechanisms and to which extent the antiapoptotic and antiproliferative effects observed in our canine endothelial cell culture model could also apply to the *in vivo* situation. 

## Abbreviations

bFGF: Basic fibroblast growth factor
CnAoECs: Canine aortic endothelial cells
CECBM: Canine endothelial cell basal medium
CECGM: Canine endothelial cell growth medium
FFA: Free fatty acid
FN: Fibronectin
GSH: Glutathione
HBSS: Hank's Buffered Salt Solution
ALenS: *γ*-Linolenic acid
LolS: Linoleic acid
NAc: N-Acetylcysteine
Pio: Pioglitazone
Rosi: Rosiglitazone
SD: Standard deviation
VEGF: Vascular endothelial growth factor.
